# Cloning and function analysis of *ZmICE1a*, a contributor to the melioration of maize kernel traits

**DOI:** 10.1080/15592324.2025.2521320

**Published:** 2025-07-02

**Authors:** Yifei Xiao, Liang Tu, Yulin Jiang, Pengfei Liu, Xiangyang Guo, Angui Wang, Yunfang Zhu, Xuefeng Lu, Zehui Chen, Xun Wu

**Affiliations:** a College of Agriculture, Guizhou University, Guiyang, Guizhou, China; b Institute of Upland Food Crops, Guizhou Academy of Agricultural Sciences, Guiyang, Guizhou, China; c Ministry of Agriculture and Rural Affairs Key Laboratory of Crop Genetic Resources and Germplasm Innovation in Karst Region, Guiyang, Guizhou, China

**Keywords:** Maize, *ZmIce1a*, kernel trait, overexpression

## Abstract

Kernel traits are important factors in determining maize yield. Gene mining and clarification of relevant gene functions associated with kernel traits is beneficial for breeding high-yield maize varieties. In our previous research, a critical quantitative trait locus (QTL), *qKWEI3.1*, associated with kernel weight was mapped using a maize F_2:3_ population derived from the parental lines SCML0849 and ZNC442. In the present study, *qKWEI3.1* was fine-mapped, the *ZmICE1a* gene was cloned, and the relevant functions of *ZmICE1a* were dissected. The results showed that plants overexpressing *ZmICE1*a exhibited a shorter reproductive period, increased plant height, greater stem diameter, higher photosynthetic efficiency, and meliorated kernel traits. Transcriptome analysis revealed that *ZmICE1a* overexpression mediated differentially expressed genes (DEGs) such as *SNRK2–10*, the E3 ubiquitin-protein ligase *AIP2*, *Pho1*, and *Pho2*. Gene ontology and KEGG pathway enrichment analyses revealed that the DEGs were involved in the abscisic acid signaling pathway, starch, and sucrose metabolism. These results suggest that *ZmICE1a* is a critical, positive regulator promoting plant growth and meliorating kernel traits. The findings of this study have important implications for the improvement of grain yield through the application of genetic engineering in maize breeding.

## Introduction

Maize is a major food crop grown worldwide. The extensive application of maize in animal husbandry, as an industrial raw material, and in the food-processing industry underscores its importance for global food security and economic development.^1^ Consequently, boosting maize yields is crucial for enhancing human quality of life and remains a primary objective of current maize breeding programs.^2^ Maize kernel traits are important contributors to yield, including kernel length and width, 100-kernel weight, protein content, and starch content.^3^ Clarification of the genetic and developmental mechanisms of kernel traits is beneficial for the rapid development of new high-yielding varieties.^4^

In recent decades, many genetic loci and candidate genes associated with kernel traits have been reported by mapping quantitative trait loci (QTLs) or through genome-wide association studies (GWAS).^5,6^ Liu et al.^7^ identified 12 QTLs associated with maize kernel size and weight, accounting for 10% of the total phenotypic variation. Zhang et al.^8^ identified 19 common QTLs and 18 significant single-nucleotide polymorphisms (SNPs) associated with maize kernel size. Liu et al.^9^ identified a critical gene, *ZmINCW1*, associated with kernel size and weight. Further research showed that overexpression of *ZmINCW1* reduces seed weight.^9^ Map-based cloning revealed that one kernel size-related gene, *qwk9*, encodes a pentatricopeptide repeat protein, and influences photosynthesis and grain filling.^10^

In addition, many genes associated with maize kernel development have been identified using homology searches or mutant-based cloning.^10^ Using the maize kernel mutant *defective kernel 42*, the *dek42* gene was cloned and shown to significantly disrupt the expression of thousands of genes during kernel development, resulting in small, defective kernels.^11^ Utilizing a different maize small-kernel mutant, the *smk501* gene was cloned and shown to encode a RUBylation-activating enzyme E1 subunit *ZmECR1* (*E1 C-TERMINAL RELATED 1*) protein, and affected kernel development by regulating AUX/IAA degradation.^12^ These genes cloned from mutants usually have critical functions in important developmental processes and may cause lethality. Thus, it is difficult to utilize these genes effectively in breeding. Therefore, gene mining by map-based cloning and investigation of weak mutations remains invaluable.

In our previous research, we mapped a critical QTL, *qKWEI3.1*, associated with maize kernel weight using a population of F_2:3_ progeny derived from the parental lines SCML0849 and ZNC442.^13^ In the present study, 419 SNPs located within 8 Mb adjacent to this QTL were used to narrow the genetic location and clone a candidate gene for *ZmICE1a* (*ZmbHLH175*). Recently, Wang Q et al.^14^ generated loss-of-function mutants of *ZmICE1a* using CRISPR/Cas9 and found that *ZmICE1a* plays a crucial regulatory role in the endosperm defense response and coordinates the balance between defense and storage during endosperm development through the JA-*ZmJAZ9-ZmICE1a*-MPI signaling pathway. Coincidentally, in our research, the functions of *ZmICE1a* were dissected by integrating overexpression (OE) and transcriptome analysis. The objectives of the study were as follows: 1) fine map the QTL *qKWEI3.1*; 2) dissect the positive function of *ZmICE1a* in meliorating maize kernel width, 100-weight, and starch content; 3) elucidate the function of *ZmICE1a* in the early reproductive phase, and in increasing the plant height, stem diameter, and photosynthetic efficiency; and 4) analyze the regulatory network of *ZmICE1a* and other genes involved in kernel development. The results provide an important genetic basis for the improvement of yield in maize breeding programs.

## Materials and methods

### Plant materials

For fine mapping of the QTL *qKWEI3.1*, a population of F_2:3_ lines derived from the parental lines SCML0849 and ZNC442 was used.^13^ For function analysis of *ZmICE1a*, the maize inbred lines B104 were used as the wild type (WT) and OE lines were generated from the B104 inbred line.

### Fine mapping of the QTL qKWEI3.1

In this study, one F_2:3_ population comprising 133 lines was selected. A total of 410 SNP markers located within an 11 Mb interval adjacent to *qKWEI3.1* were selected (Table S2). QTL analyses of F_2:3_ for the interval were conducted using inclusive composite interval mapping (ICIM)in QTL IciMapping software Version 4.1.^15^ Using the B73 reference genome (version 4), a bioinformatics analysis was conducted by utilizing the public database MaizeGDB (https://www.maizegdb.org.) to identify candidate genes.

### Cloning of ZmICE1a

Total RNA was extracted using the TRIzol Reagent (Takara, Dalian, China) and treated with DNase to remove contaminant genomic DNA. The PrimeScript™ 1st Strand cDNA Synthesis Kit (Takara) was used for the cDNA synthesis in accordance with the manufacturer’s instructions. Primers were designed with SnapGene® 6.0.2 (GSL Biotech LLC, San Diego, CA, USA) based on the maize B73 reference genome V4 accessed in MaizeGDB (https://www.maizegdb.org.) (Table S1). The CDS sequences of *ZmICE1a* from B73, B104, ZNC442, and SCML0849 were cloned. The PCR products were verified by 1% agarose gel electrophoresis and purified using the DNA Gel Purification Kit (UElandy, Suzhou, China). The purified target fragment was cloned into *Escherichia coli* strain DH5α cells using the Lethal Based Simple Fast Cloning Kit (Tiangen Biotech, Beijing, China). Single colonies were verified by colony PCR and authenticated by 1% agarose gel electrophoresis, then positive clones were sequenced by Sengon Biotech (Shanghai, China).

### Genetic structure analysis of ZmICE1a

The CDS sequences of *ZmICE1a* from B73, B104, ZNC442, and SCML0849 were first aligned using SnapGene® 6.0.2 (GSL Biotech LLC, San Diego, CA, USA), and their deduced amino acid sequences were compared. The coding sequence (CDS) of *ZmICE1a* cloned from B104 was used as the query in a BLAST search of the National Center for Biotechnology Information databases (NCBI; https://www.ncbi.nlm.nih.gov/.) and *Nicotiana benthamiana* and *Nicotiana tabacum* omics databases (Nicomics) to detect homologs in other plant species, including rice, wheat, Arabidopsis, *Nicotiana tabacum*, and so on. The obtained sequences were aligned using the ClustalW algorithm and a phylogenetic tree was constructed with MEGA Version 11 using the Neighbor-joining model. Sequence motif analysis was performed using the online MEME tool (https://meme-suite.org). Visualization of the phylogenetic tree and motif analysis was amalgamated using ChiPlot (https://www.ChiPlot.online). The 3000 bp sequence upstream of the *ZmICE1a* promoter was extracted from B73 reference genome V4 accessed in MaizeGDB (https://www.maizegdb.org.) and the genetic structure was predicted using the PlantCARE database (https://bioinformatics.psb.ugent.be/webtools/plantcare/.).

### Subcellular localization

To further evaluate the function of the protein encoded by *ZmICE1a*, the subcellular localization of ZmICE1a was assessed. The CDS of *ZmICE1a* was inserted into the PCAMBIA2300-35S-*eGFP* vector. The construct and the empty vector were transiently expressed in maize protoplasts and tobacco leaves, using a helium biolistic gun transformation system (Bio-Rad, Hercules, CA, USA). The GFP signal was visualized and captured using a confocal laser scanning microscope (Leica TCS SP5, Mannheim, Germany) with excitation at 488 nm.

### Expression pattern analysis of ZmIce1a

For the B104 inbred line, different tissues were collected at four developmental stages, namely, jointing (V8), booting (V12), tasseling (VT), and grain filling (R2), based on a previous description of growth stages.^16^ Total RNA was extracted and cDNA was synthesized as described above. Quantitative real-time PCR (qPCR) was used to determine the expression level of *ZmICE1a*, using *ZmACTIN2* as the internal reference gene, for three independent biological samples. All primers were designed using the PrimerQuest Tool (https://sg.idtdna.com/pages.) (Table S1). The fold difference (2^−∆∆*C*t^) and relative expression levels were calculated using CFX Manager Software v1.5 (Bio-Rad).

### Generation of ZmIce1a-overexpressing plants

The cDNA fragment of *ZmICE1a* was submitted to Wimi Biotechnology Co., Ltd. (Changzhou, China). The cDNA fragment was inserted downstream of the ubiquitin promoter to construct transgenic lines. Thereafter, the company conducted a series of meticulous experimental procedures to generate T1 seeds as follows. First, primers were designed based on the vector and the *ZmICE1a* cDNA template sequence. The upstream primer F combined the upstream 16 bp of the *ZmICE1a* cDNA template with the 16 bp upstream recombination arm, while the downstream primer R combined the downstream 16 bp of the template with the 16 bp downstream recombination arm.

Next, a 2×Rapid Taq Master Mix (Vazyme, Nanjing, China) was implemented in a 50 μL PCR reaction system. After completion of the PCR reaction, 3 μL of the product was analyzed by electrophoresis to verify the amplification of the correct fragment. If the correct band was observed, the target fragment was recovered using the FastPure Gel DNA Extraction Mini Kit DC301 kit (Vazyme, Nanjing, China) stock solution. The recombinant plasmid was transformed into *E.coli* DH5α cells and validated through colony PCR sequencing. Upon confirming the sequence accuracy, the vector plasmid was introduced into *Agrobacterium tumefaciens* strain EHA105 via electroporation and further identified by PCR.

For the transformation, freshly dissected immature maize embryos, approximately 1 mm in size, were used. The isolated maize embryos were placed in a 2 mL plastic centrifuge tube containing 1.8 mL of infection buffer, with approximately 150 immature embryos processed over a 30-min period. The infection buffer was removed, then 1.0 mL *Agrobacterium* suspension was added and the mixture was incubated for 5 min. The embryos were gently poured onto the co-culture medium, and any excess *Agrobacterium* suspension on the surface was removed. The embryos and *Agrobacterium* were co-cultured in the dark at 23°C for 3 days. Then, the embryos were transferred to a resting medium and incubated in the dark at 28°C for 6 days. The embryos were then transferred to a screening medium supplemented with dialaphos for an initial 2-week screening, then incubated for an additional 2 weeks on a fresh screening medium. The antibiotic-resistant calli were transferred to a differentiation medium and cultured at 25°C under a light intensity of 5000 l×for 3 weeks. The differentiated seedlings were subsequently transferred to the rooting medium and incubated under the same environmental conditions until roots formed. The rooted seedlings were transplanted into small pots for further growth and subsequently transferred to the greenhouse. After growth for 3–4 months, seeds were harvested for subsequent analysis.

The T_0_ transgenic plants were genotyped using primers for the marker gene in the vector (Table S1). The expression level of *ZmICE1a* in positive transgenic lines was assessed by qPCR as described above. After repeated selfing, three homozygous T_3_ lines were selected and designated *ZmICE1a*-overexpressing lines 1, 2, and 3 (hereafter OE1, OE2, and OE3). Uniform, healthy kernels from the three T_3_ OE lines and the relevant WT (B104) were grown in an experimental field at Le Dong (LD; 18.24°N, 108.39°E, Hainan Province, China) from October 2023 to March 2024, and cultivated in greenhouses located in Gui Yang (GY; 26.11°N, 106.7°E, Guizhou Province, China) from March 2024 to August 2024. Phenotypic analyses were carried out at both locations.

### Phenotypic analysis of ZmIce1a-overexpressing plants

Phenotypic traits for the OE lines and WT were recorded, comprising the stem size of plants at the V8 stage, plant height, days to tasseling (DTT), days to pollen shedding (DTP), days to silking (DTS), and chlorophyll content were measured at the VT stage. Each trait was measured on five plants. Mature grains were harvested and dried, and then kernel traits, including the kernel weight, length, and width, were measured using the SC-G software (Wanshen Detection Technology Co., Ltd., Hangzhou, China). Twenty uniform kernels were randomly selected, and submerged in a 100 ml graduated cylinder containing 20 ml water, and the change in water level was recorded to calculate the individual kernel volume. The maximum quantum efficiency of photosystem II (*F*_V_/*F*_M_) of ear leaves was estimated using an OS30p+ Chlorophyll Fluorometer (OPTI-SCIENCES Inc., Hudson, NH, USA). All photosynthesis measurements were performed with three biological replicates.

### Starch content assay

The starch content of kernels from *ZmICE1a*-overexpressing and WT plants was determined as described by Clegg^17^ using the Starch Content Assay Kit (Solarbio Science and Technology, Beijing, China) following the manufacturer’s instructions. For each OE line and the WT, measurements were performed with three biological replicates. The experimental samples and standard samples from the Starch Content Assay Kit were analyzed using a microplate reader. The standard curve generated from the standard samples is presented in Table S2. Subsequently, the starch content was calculated according to the provided formulas.

### Transcriptome analysis of ZmIce1a-overexpressing plants

To elucidate the regulatory network involving *ZmICE1a*, transcriptome analysis was performed using RNA sequencing (RNA-Seq). The ear leaves and kernels in central ears were sampled from WT, OE1, and OE2 plants grown in the greenhouse. Samples from three individual plants were collected 14 days after pollination and pooled. Transcriptome analysis was performed by JiYu Technology Co., Ltd. (Chengdu, China). Adapter sequences and low-quality reads were filtered using FastQC (http://www.bioinformatics.babraham.ac.uk/projects/fastqc/). Subsequently, the cleaned reads were mapped to the maize Zm-B73-REFERENCE-NAM-5.0 reference genome using HISAT. Differentially expressed genes (DEGs) were identified by comparing the gene expression levels between the OE and WT samples. The DEGs were screened using the following criteria: fold change ≥ 2.00 and *p*-value ≥0.8, with a significant false discovery rate-adjusted *p*-value <0.05 based on three biological replicates. The identified DEGs were subjected to Gene Ontology (GO) and Kyoto Encyclopedia of Genes and Genomes (KEGG) pathway enrichment analyses using the clusterProfiler version 3.8 R package and the OmicShare online tool (https://www.omicshare.com/tools/).^18^ The transcriptome data were visualized with the OmicShare (https://www.omicshare.com/tools.) and ChiPlot (https://www.ChiPlot.online) online tools.

### Statistical analysis

One-way analysis of variance was conducted using GraphPad Prism 9.0 (GraphPad, San Diego, CA, USA) to perform multiple comparisons.^19^ A significance level was established consistent with the *New England Journal of Medicine* policy for *P*-values.^20^ Student’s *t*-test was employed for pairwise comparisons with the significance threshold set to *p* < 0.05. All presented data are mean values derived from independent experiments.

## Results

### Fine mapping of the QTL qKWEI3.1

**Figure 1.**
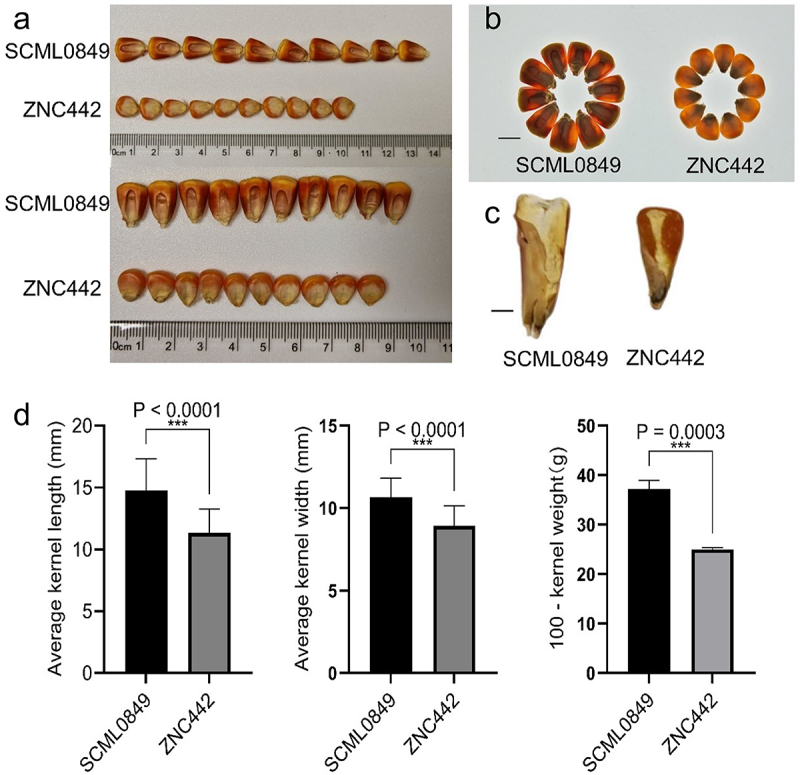
The kernel relative traits of ZNC442 and SCML0849 inbred lines. (a) The kernel pictures of ZNC442 and SMCL0849. (b) Kernel phenotypes observed on a lightbox ZNC442 and SMCL0849. Bar=10 mm. (c) Longitudinal sections of ZNC442 and SMCL0849. Bar=2 mm. (d) Kernel relative traits of ZNC442 and SMCL0849 including Average kernel length, Average kernel width, 100-kernel weight.

**Figure 2.**
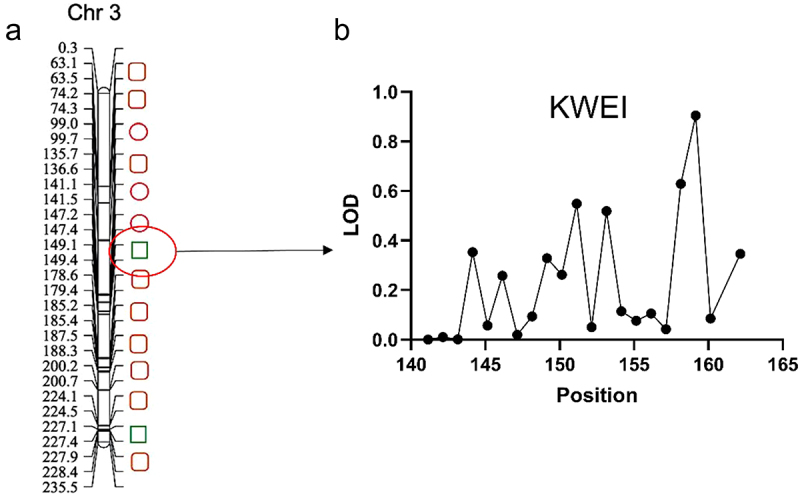
Fine Mapping and Candidate Gene Identification for Maize Kernel Weight (KWEI) on Chromosome 3 (Chr 3). (a) Red circle represents markers used in the first-round mapping, identifying a 0.3 Mb interval containing *qKWE3.1*.^13^ (b) Green square represents KWEI.

Using an F_2:3_ population derived from parents with contrasting kernel phenotypes (*large-kernel floury* inbred SCML0849 × *small-kernel flint* inbred ZNC442) (Figure 1), our prior work mapped qKWE3.1—a major QTL controlling 100-kernel weight in maize – to a 0.3 Mb interval on chromosome 3 (Figure 2(a)).^13^ Fine-mapping was performed using 410 additional markers spanning an extended region (141–162 Mb). High-resolution linkage analysis resolved a distinct peak LOD score at 159.1419 Mb (Figure 2(b); Table S3). Screening of candidate genes within ± 400 kb of this peak identified *ZmICE1a* as the only gene with known functional relevance to kernel development (Table 1). Based on its positional priority and functional annotation, *ZmICE1a* was prioritized for functional validation.

**Table 1.** Candidate genes in the interval.

ID	Description
Zm00001d042263	bHLH transcription factor, own-regulates starch synthesis
Zm00001d042264	Unknow
Zm00001d042265	Unknow
Zm00001d042266	Unknow
Zm00001d042267	ARF-transcription factor 10
Zm00001d042268	*coryne1*, involved in shoot apical meristem (SAM) development
Zm00001d042269	*pza00827*, DNAJ heat shock N-terminal domain-containing protei
Zm00001d042270	Unknow
Zm00001d042271	Trihelix-transcription factor 30,
Zm00001d042272	putative histone-lysine N-methyltransferase family protein, candidate gene for kernel number per row
Zm00001d042273	Unknow
Zm00001d042274	*cl615_-1*, phosphatidylinositol transfer protein CSR1
Zm00001d042275	*cl615_-3*, phosphatidylinositol transfer protein CSR1
Zm00001d042276	putative beta-14-xylosyltransferase IRX10L
Zm00001d042277	*PAP1*, inositol polyphosphate multikinase IPK2-like
Zm00001d042278	Unknow
Zm00001d042279	*beta-1*,4-xylosyltransferase4
Zm00001d042280	Unknow
Zm00001d042281	*beta-*1,4-xylosyltransferase2
Zm00001d042282	Aldolase-type TIM barrel family protein
Zm00001d042283	Unknow
Zm00001d042284	*pco085637a*, RING/U-box superfamily protein

### Cloning and sequence analysis of the candidate gene ZmIce1a

Gene sequence analysis confirmed *ZmICE1a* CDS presence in all four inbred lines (Figure S1A), but revealed critical structural and functional divergences: B73 exhibited a 40bp CDS deletion (Figure S1A). SCML0849 and B104 showed 100% identical CDS sequences with zero variations across the entire coding region (Supplementary Figure S2). ZNC442 harbored six non-synonymous mutations in its CDS – specifically 300(G→C), 678(C→T), 699(G→A), 757(C→T), 874(T→G), and 979(G→A)(Supplementary Figure S2) – resulting in four amino acid substitutions compared with SCML0849: 114(Alanine→Proline), 253 (Phenylalanine→Leucine), 292(Alanine→Serine) and 327 (Leucine→Valine) compared with SCML0849 (Supplementary Figure S3).

**Figure 3.**
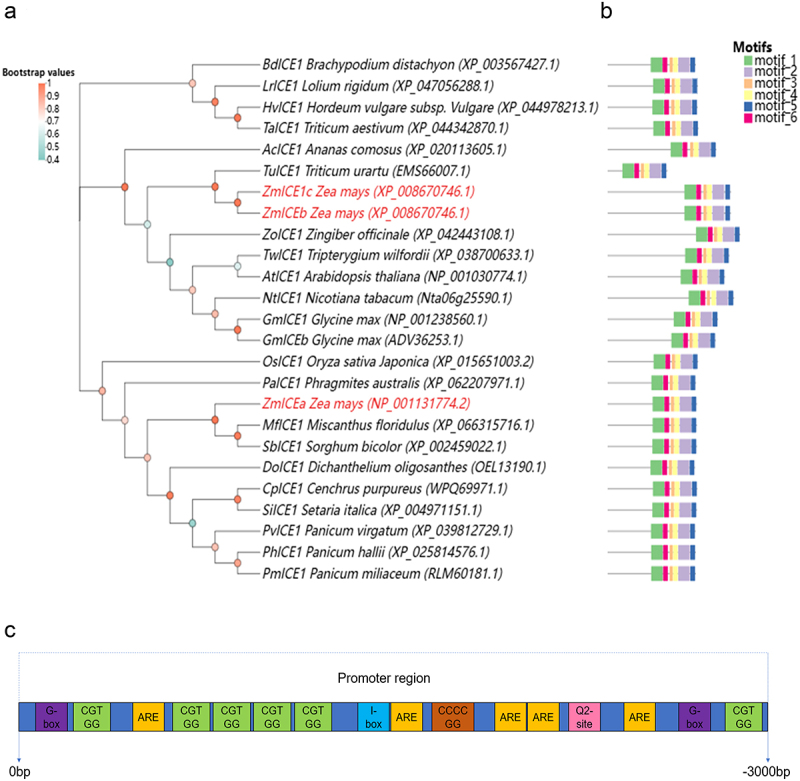
Bioinformation analysis of *ZmICEa*. (a) Phylogenetic analysis of *ICE1* homologous genes from rice, tobacco, Arabidopsis, wheat, and so on. (b) Prediction of motifs in *ICE1* homologs. (c) Identification of cis-elements in the promoter region of *ZmICE1a*. The color blocks indicated the different cis-elements (Purple: G-box; Green: CGTGG; Yellow: ARE; Baby blue: I-box; Brown: CCCCGG; Pink: O_2_-site).

Sequence alignment and phylogenetic analysis indicated that *ZmICE1a* was clustered on a separate branch alone and had a relatively close phylogenetic relationship with *sorghum* and *Miscanthus floridulus*. Meanwhile, *ZmICE1b* and *ZmICE1c* were clustered together, and their phylogenetic affinity was closer to *Triticum Urartu*. Furthermore, *ZmICE1b* and *ZmICE1c* were grouped into a large category with dicotyledonous plants such as *Arabidopsis thaliana*, *Nicotiana tabacum*, and *Glycine max*. (Figure 3(a)). Motif analysis revealed that homologs of *ZmICE1a* are extremely conserved across monocotyledonous plants and share most of the same motif location. While the motifs of *ZmICE1b* and *ZmICE1c* are extremely similar. (Figure 3(b)). These results suggest that definite evolutionary disparities and divergences exist between *ZmICE1a* and *ZmICE1b/ZmIC1Ec* and Zm*ICE1a* may perform similar functions in monocotyledonous plants.

The 3000 bp sequence upstream of *ZmICE1a* was extracted to analyze the structure of the *ZmICE1a* promoter. Six specific *cis*-acting regulatory elements were identified, comprising I-box, G-box, AU-rich (ARE), and O2-site elements, and two unnamed elements, CGTGG and CCCCGG (Figure 3(c) and Table S4). The I-box and G-box elements are involved in light response.^21,22^ The ARE sequence is a *cis*-acting regulatory element essential for anaerobic induction.^23^ The O2-site is involved in the regulation of zein metabolism, which is a major constituent of the maize endosperm.^24^ These results suggest that *ZmICE1a* may be involved in light response, antimicrobial interaction, and seed development in maize.

### Subcellular localization of ZmIce1a

**Figure 4.**
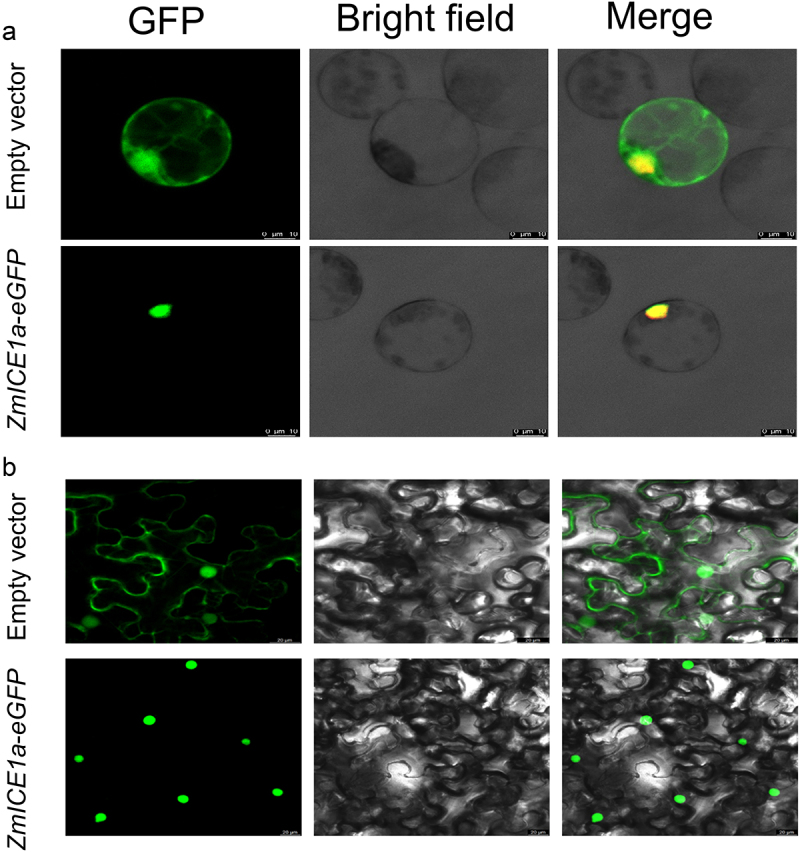
Subcellular localization of *ZmICE1a*. A-B Subcellular localization of *ZmICE1a-eGFP* in maize protoplasts (a) and Subcellular localization of tobacco leaf epidermal cells (b). GFP: Green Fluorescent signal; RFP: Red Fluorescent signal. Scale bars = 10 μm in (a) and 20 μm in (b).

Subcellular localization analysis showed that the fusion protein *ZmICE1a-eGFP* was detected only in the nucleus in maize protoplasts and tobacco leaf cells, whereas the fluorescence signal of the empty vector was detected throughout the protoplast (Figure 4). Therefore, it can be concluded that *ZmICE1a* may function in the nucleus, consistent with the function of a transcription factor.

### Expression pattern of ZmIce1a

**Figure 5.**
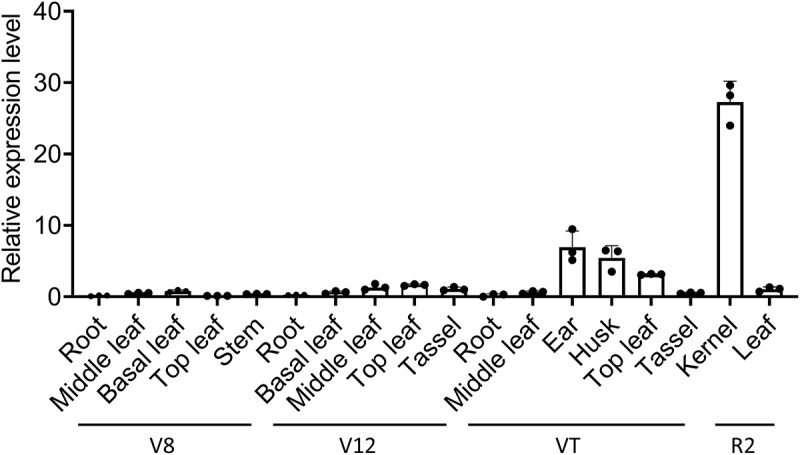
The relative expression levels of *ZmICE1a* in different tissues at V8, V12, VT, and R2 stages.

The expression levels of *ZmICE1a* varied significantly among tissues at various maize developmental stages (Figure 5). Notably, *ZmICE1a* was highly expressed in the kernel tissue at the R2 stage when kernels began to form. These results suggest that *ZmICE1a* is closely associated with maize growth and development, and may play important roles in kernel formation.

### Phenotypic analysis of ZmICE1a-overexpressing and WT plants

**Figure 6.**
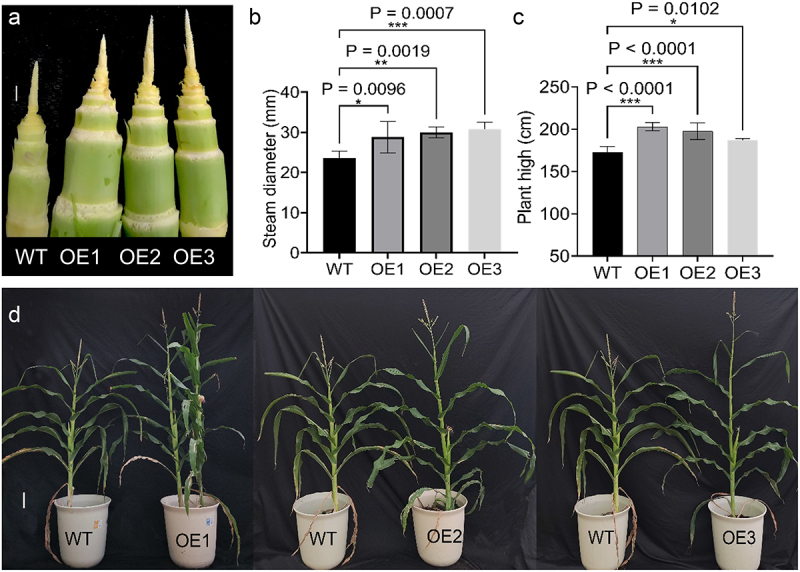
The phenotypes of OE lines and WT plants. (a) The maize internodes OE plants after seeding 60 days. Bar=1 cm. (b) The steam diameter of OE plants after seeding 60 days. (c) The plant height of mature OE plants. (d) The pictures of different OE lines at the VT stage. Scale bars = 1 cm in (a) and 10 cm in (d). The significant differences are determined by Dunnett’s Multiple Comparisons Test and marked with NEJM (New England Journal of Medicine) style of P-value (* P<0.033, ** P<0.002, and *** P<0.001).

**Figure 7.**
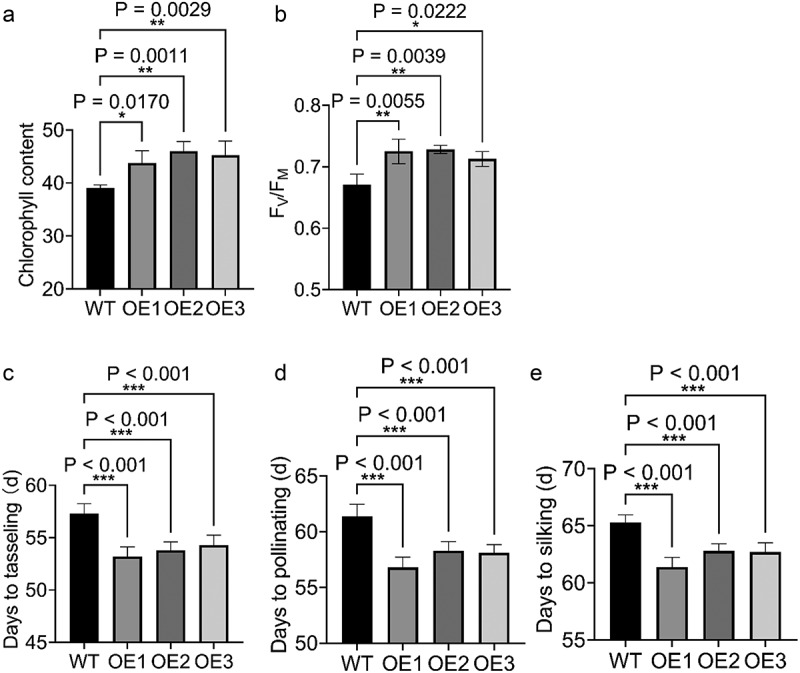
The index of photosynthetic efficiency and flowering-time traits of WT and OE lines. The chlorophyll content (a) and the F_V_/F_M_ (b) of ear leaves. c-e The flowering-time traits including the days to tasseling (c), the days to pollinating (d), and the days to silking (e). The significant differences are determined by Dunnett’s Multiple Comparisons Test and marked with NEJM (New England Journal of Medicine) style of P-value (* P<0.033, ** P<0.002, and *** P<0.001).

Among a total of 12 lines of T_0_ plants, 10 positive transgenic lines were identified (Fig. S4A). Relative expression levels were assessed using the qPCR method. Expression levels of *ZmICE1a* in OE1, OE2, and OE3 plants were significantly higher than those of WT plants (Fig. S4B, C). In addition, T_0_ plants of the OE1 and OE2 lines produced higher biomass than WT plants (Fig. S4D). Plants of the OE1, OE2, and OE3 lines were repeatedly selfed to generate T_3_ homozygous lines. Phenotypic analysis of the T_3_ homozygous lines was conducted in both field and greenhouse environments. Compared with WT plants, the T_3_ OE lines exhibited a more robust phenotype, such as enhanced aboveground architecture (Fig. S4D), thicker stems (Figure 6(a,b)), and an increase in plant height (Figure 6(c,d)). Given that *ZmICE1a* was predicted to be involved in light response (Figure 3(c)), we wondered if the stronger plants reflected higher photosynthetic efficiency in the OE lines. The chlorophyll content and *F*_V_/*F*_M_ in ear leaves of the OE lines were significantly higher than those of WT plants (Figure 7(a,b)), suggesting that the higher chlorophyll content might account for the increase in photosynthetic efficiency. Consequently, we concluded that overexpression of *ZmICE1a* enhanced the photosynthetic efficiency, resulting in a more robust phenotype compared with that of WT plants.

Furthermore, the OE plants grew more rapidly and entered the reproductive phase earlier than WT plants. Evaluation of flowering-time traits showed that DTT, DTS, and DTP of OE plants were less than those of WT plants (Figure 7(c–e)). These results indicated that overexpression of *ZmICE1a* accelerated the transition from vegetative to reproductive growth, and thus *ZmICE1a* might be associated with auxin mediation of this developmental change.

### Overexpression of ZmIce1a meliorates kernel traits of maize

**Figure 8.**
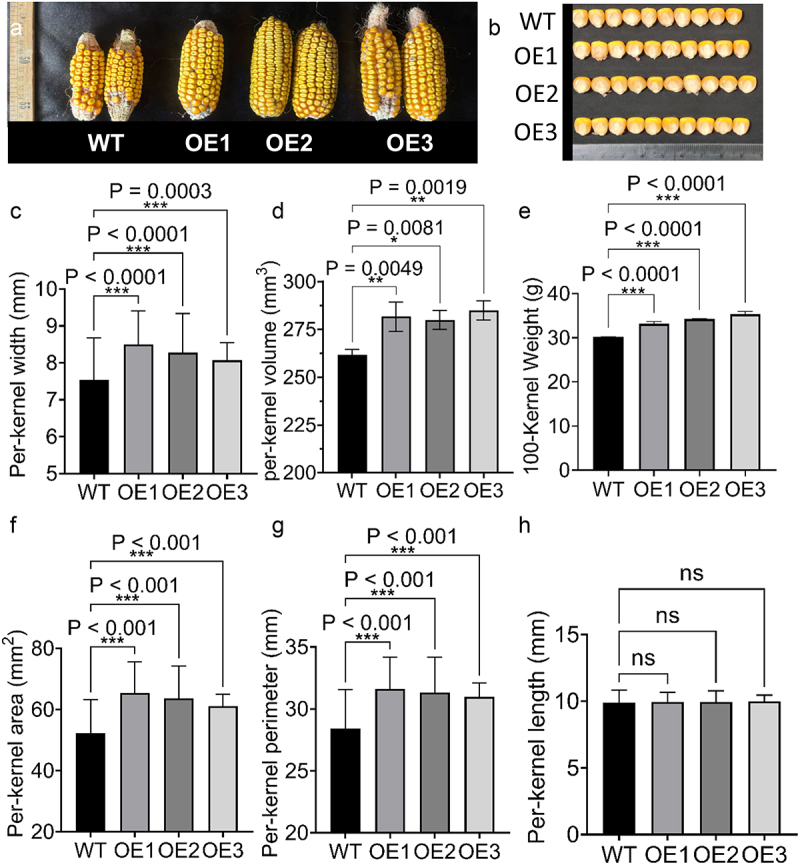
The ear phenotypes and kernel relative traits of WT and OE lines. (a) The ear pictures of different OE lines. (b) The kernel pictures of OE lines. c-g Kernel relative traits of OE lines including per-kernel width (c), 1000-kernel weight (d), per-kernel volume (e), per-kernel area (f), per-kernel perimeter (g), and per-kernel length (h).

**Figure 9.**
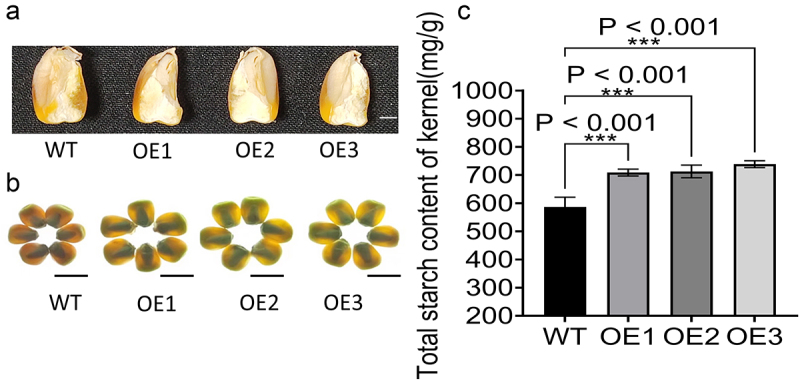
Starch concentration analysis of kernels. (a) Longitudinal section of OE lines. Bar=2 mm. (b) Kernel phenotypes observed on a lightbox. Bar=10 mm. (c) Total starch content of kernel of OE lines. The significant differences are determined by Dunnett’s Multiple Comparisons Test and marked with NEJM (New England Journal of Medicine) style of P-value (* P<0.033, ** P<0.002, and *** P<0.001).

Mature ears of OE and WT plants growing in the field and greenhouse were harvested. Compared with WT plants, OE plants had larger ears and kernels (Figure 8(a,b)). Statistical analysis of the kernel traits revealed that kernel width, 100-kernel weight, kernel volume, kernel area, and kernel perimeter (Figure 8(c–g)) were significantly increased compared with those of WT plants, while there was no significant difference in kernel length. (Figure 8(h)). In addition, the OE plants’ kernels were floury than those of the WT (Figure 9(a,b)), suggesting that the kernels may have a higher starch content. As predicted, the total starch content was significantly higher in OE plants than in the WT (Figure 9(c)). Considering the foregoing results, we concluded that overexpression of *ZmICE1a* may promote maize plant growth by improving photosynthetic efficiency, thereby meliorating kernel traits.

### Functional enrichment analysis of differentially expressed genes

**Figure 10.**
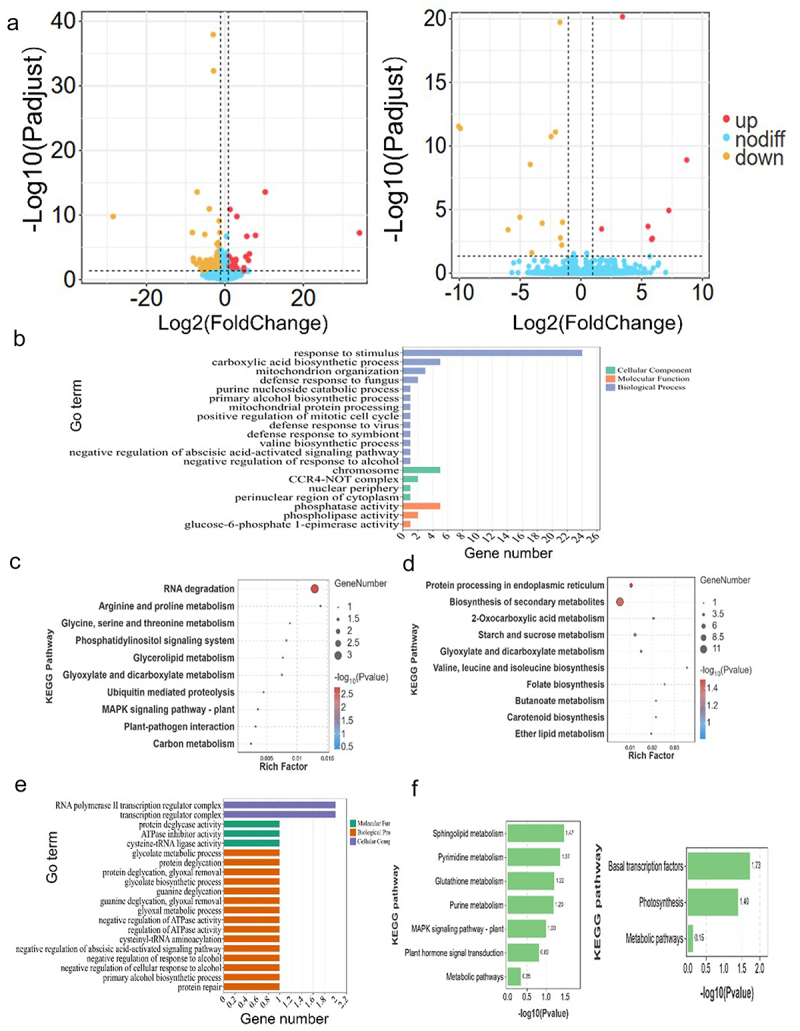
Functional enrichment analysis of differentially expressed genes (DEGs) in the ear leaf and kernel of OE lines collected on the 14^th^ day after pollination when compared with WT. (a) DEGs numbers in kernels (top) and ear leaves (bottom). (b) Gene Ontology (GO) annotation of DEGs in kernels. c-d Kyoto Encyclopedia of Genes and Genomes (KEGG) enrichment analysis of upregulated (c) and downregulated (d) genes in kernels. e-f GO annotation (e) and KEGG analysis (f) of DEGs in ear leaves.

To dissect the molecular mechanisms underlying *ZmICE1a* function, we employed RNA-Seq to analyze the transcriptome of developing kernels and ear leaves. A total of 141 DEGs were identified in developing kernels of *ZmICE1a*-overexpressing plants, compared with those of the WT, of which 19 and 122 genes were up-regulated and down-regulated, respectively (Figure 10(a) and Table S5). In contrast, 20 DEGs were detected in the ear leaves comprising seven up-regulated genes and 13 down-regulated genes (Figure 10(a) and Table S5). A total of 20 GO terms were enriched in developing kernels, which were mainly categorized into response to stimulus, carboxylic acid biosynthetic process, phosphatase activity, and chromosome organization (Figure 10(b) and Table S6). To further predict metabolic pathways, we performed a KEGG pathway enrichment analysis. Up-regulated DEGs in developing kernels were notably enriched in the RNA degradation pathway (Figure 10(c); Table S7). Down-regulated DEGs were especially associated with protein processing in the endoplasmic reticulum, biosynthesis of secondary metabolites, 2-oxocarboxylic acid metabolism, and starch and sucrose metabolism (Figure 10(d) and Table S7), suggesting that these signaling pathways involving *ZmICE1a* may contribute to maize kernel development.

Concerning the ear leaf, DEGs were mainly involved in the RNA polymerase II transcription regulator complex, transcription regulator complex, ATPase inhibitor activity, and negative regulation of the abscisic acid-activated signaling pathway (Figure 10(e) and Table S6). KEGG pathway enrichment analysis showed that up-regulated genes were significantly enriched in the sphingolipid metabolism and pyrimidine metabolism pathways, whereas down-regulated genes were predominantly associated with basal transcription factors and photosynthesis (Figure 10(f) and Table S7), suggesting that these pathways may enhance the photosynthetic efficiency of the OE plants.

**Figure 11.**
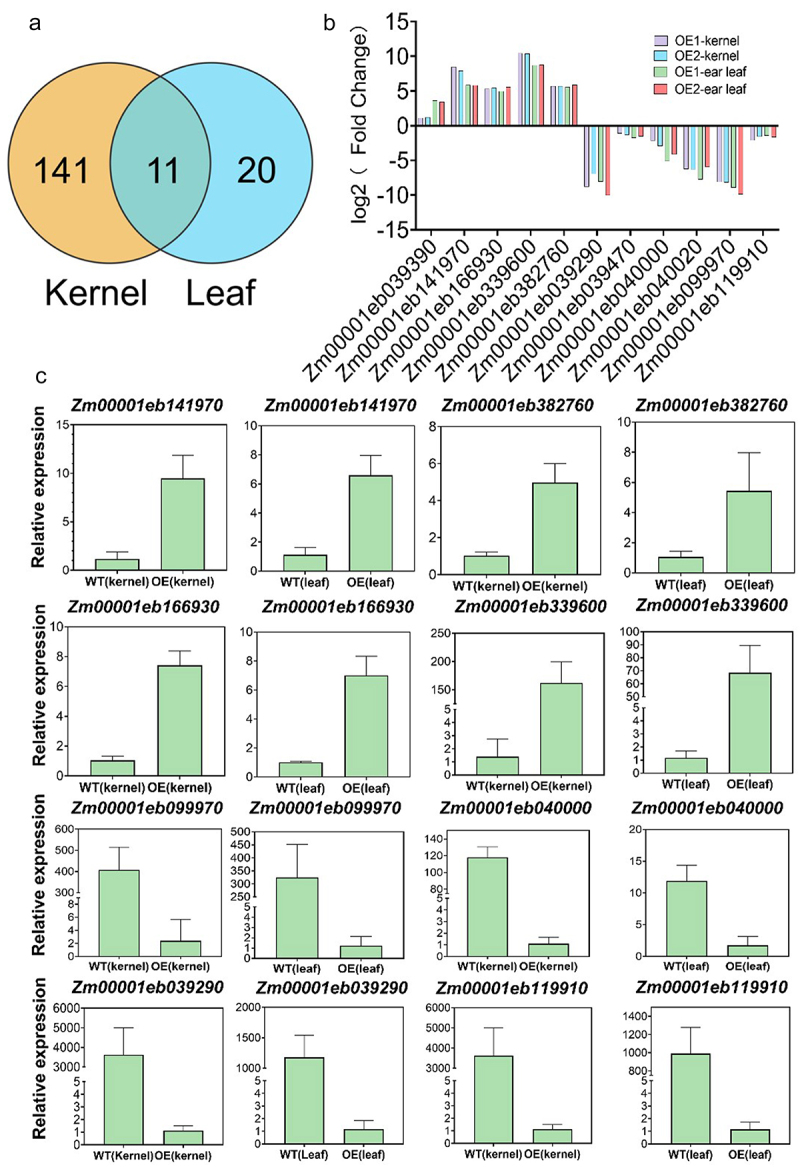
Common DEGs analysis in the kernels and ear leaves. (a) Common DEGs number in the kernel and ear leaf. (b) Relative expression levels of common DEGs. (c) KEGG enrichment analysis of common DEGs. (d) The verification of relative expression levels of DEGs by RT-qPCR. The significant differences are determined by the Student’s *t*-test (P<0.001).

Eleven DEGs were co-expressed in both developing kernels and ear leaves (Figure 11(a) and Table S8). These genes were implicated in stress-induced mitochondrial fusion (*Zm00001eb039290*), a serine proteinase inhibitor (*Zm00001eb040020*), RING-like zinc finger protein (*Zm00001eb099970*), a DJ-1/PfpI family member (*Zm00001eb119910*), mitochondrial ATPase inhibitor (*Zm00001eb039390*), a member of the protein kinase superfamily (*Zm00001eb141970*), two members of the low-molecular-weight phosphatase family (*Zm00001eb339600*, *Zm00001eb382760*) and unannotated genes (Zm00001eb039470, Zm00001eb040000, Zm00001eb119910) (Table S8). Notably, *Zm00001eb141970*, synonymous with the serine/threonine-protein kinase *SAPK3*, was up-regulated in both kernels and leaves (Figure 11(b, c)). The *SnRK2* family, also known as the *SAPK* (*SnRK2*-Associated Protein Kinase) family, is pivotal in the abscisic acid (ABA) signaling pathway.^25^ Intriguingly, Lou et al.^26^ demonstrated that *OsSPAK3* can positively affect grain length, tiller number, and yield in rice. Given the comprehensive phenotypic data, we propose that *Zm00001eb141970* may play a pivotal role within the regulatory network of *ZmICE1a*.

## Discussion

Maize domestication involved extensive artificial selection leading to abundant genetic modifications. Our prior work mapped qKWE3.1 and identified *ZmICE1a* (a bHLH transcription factor) as its candidate gene. While Wang et al.^14^ established that *ZmICE1a* balances endosperm defense and storage via the JA–*ZmJAZ9*–*ZmICE1a*–MPI pathway, our study reveals complementary roles in direct kernel quality regulation under non-stress conditions.

Overexpression of *ZmICE1a* significantly increased kernel size, 100-kernel weight, and starch content (Figure 8(c–g)), contrasting with smaller kernels and reduced vitreous endosperm in *ZmOpaque11* mutants (Figure 9). This phenotypic antagonism, coupled with their co-activation of *ZmYoda*^27^ Fig. S4A, B), implies a potential co-regulatory mechanism for starch accumulation distinct from stress adaptation pathways.

Transcriptome analysis revealed coordinated upregulation of starch synthesis genes in both kernels and leaves of OE lines (Fig. S4C). Notably, two starch phosphorylases—*ZmPho1* (Zm00001eb057840) and *ZmPho2* (Zm00001eb147780) – were significantly enriched (Figure 10(b-d)). These enzymes catalyze reversible glucan phosphorolysis to produce glucose-1-phosphate,^28–30^ suggesting *ZmICE1a* may regulate starch metabolism through phosphorylase activity. Additionally, genes including *SSRP1*, *SSRP2*, and *SUS1* showed parallel upregulation trends across organs (Fig. S4C), indicating a source-sink synchronization mechanism that extends beyond Wang et al.’s^14^ endosperm-centric model.

*ZmICE1a* overexpression also enhanced biomass and photosynthesis (Figure 7, S4D), potentially mediated by regulation of nitrogen uptake (e.g., *ZmSAPK3*, a kinase linked to nitrogen efficiency^31,32^ and photosynthetic components (e.g., ferredoxin-NADP reductase and E3 ligase *AIP2*; Figure 10(e, f)). These pathways were previously unassociated with *ZmICE1a*, providing a mechanistic explanation for vegetative growth advantages.

Notably, no significant regulation of *ZmAS1/ZmDREB1* was detected (Table S9, Fig. S5A, B), and *ZmJAZ9* exhibited organ-specific expression trends (downregulated in leaves, upregulated in kernels; Fig. S5B). This suggests kernel developmental functions operate independently of cold-stress responses,^33,34^ consistent with prior findings.

Collectively, our work expands the functional scope of *ZmICE1a* to include:(1) Co-regulation with *ZmOpaque11* in starch biosynthesis, (2) Systemic influence on nitrogen-photosynthesis coupling, and (3) Organ-coordinated activation of starch metabolic pathways. These mechanisms highlight its potential for kernel yield improvement and illustrate how pleiotropic regulators evolve specialized functions during domestication.

## Conclusions

Using an F_2:3_ population derived from the maize parental lines SCML0849 and ZNC442, we identified a candidate gene, *ZmICE1a*, via fine-mapping of the major QTL *qKWE3.1* (previously annotated as *qKWEI3.1*, corrected for nomenclatural consistency). *ZmICE1a* as a key target for dissecting the molecular basis of maize kernel weight regulation. Bioinformatic analysis indicated that the promoter region of *ZmICE1a* contained several specific *cis*-acting regulatory elements associated with light response, antimicrobial interaction, and kernel formation. *ZmICE1a* was primarily expressed in the kernel. Overexpression of *ZmICE1a* resulted in acceleration of the transition to the reproductive phase, and increased the plant height, stem diameter, and photosynthetic efficiency, thereby meliorating kernel traits. Transcriptome analysis of three OE lines identified several DEGs associated with the ABA signaling pathway, and starch and sucrose metabolism, such as *SAPK3*, the E3 ubiquitin-protein ligase *AIP2*, *Pho1*, and *Pho2*, indicating that these genes may participate in the *ZmICE1a* regulatory pathway. The present findings highlight the important function of *ZmICE1a* in maize kernel development, providing theoretical support for a novel function, and insights for future research and breeding efforts. However, the conclusions are based on phenotypic analysis and transcriptome data for OE plants, and thus specific regulatory mechanisms and pathways require further exploration and verification.

## Supplementary Material

Supplemental Material

Supplemental Material

## Data Availability

Data will be made available on request.

## References

[1] Ray DK, Sloat LL, Garcia AS, Davis KF, Ali T, Xie W. Crop harvests for direct food use insufficient to meet the UN’s food security goal. Nat Food. 2022;3(5):367–15. doi: 10.1038/s43016-022-00504-z. 37117562

[2] Andorf C, Beavis WD, Hufford M, Smith S, Suza WP, Wang K, Woodhouse M, Yu J, Lübberstedt T. Technological advances in maize breeding: past, present and future. Theor Appl Genet. 2019;132(3):817–849. doi: 10.1007/s00122-019-03306-3. 30798332

[3] Fernández JA, Messina CD, Salinas A, Prasad PVV, Nippert JB, Ciampitti IA, Dreisigacker S. Kernel weight contribution to yield genetic gain of maize: a global review and US case studies. J Exp Botany. 2022;73(11):3597–3609. doi: 10.1093/jxb/erac103. 35279716

[4] Wang C, Li H, Long Y, Dong Z, Wang J, Liu C, Wei X, Wan X. A systemic investigation of genetic architecture and gene Resources controlling kernel size-related traits in maize. Int J Mol Sci. 2023;24(2):1025. doi: 10.3390/ijms24021025. 36674545 PMC9865405

[5] Lan T, He K, Chang L, Cui T, Zhao Z, Xue J, Liu J. QTL mapping and genetic analysis for maize kernel size and weight in multi-environments. Euphytica. 2018;214(7):119. doi: 10.1007/s10681-018-2189-0.

[6] Li C, Wu X, Li Y, Shi Y, Song Y, Zhang D, Li Y, Wang T. Genetic architecture of phenotypic means and plasticities of kernel size and weight in maize. Theor Appl Genet. 2019;132(12):3309–3320. doi: 10.1007/s00122-019-03426-w. 31555889

[7] Liu Y, Wang L, Sun C, Zhang Z, Zheng Y, Qiu F. Genetic analysis and major QTL detection for maize kernel size and weight in multi‑environments. TAG Theor Appl Genet Theoretische Angew Genetik. 2014;127(5):1019–1037. doi: 10.1007/s00122-014-2276-0. 24553962

[8] Zhang X, Guan Z, Wang L, Fu J, Zhang Y, Li Z, Ma L, Liu P, Zhang Y, Liu M. Combined GWAS and QTL analysis for dissecting the genetic architecture of kernel test weight in maize. Mol Genet Genomics. 2020;295(2):409–420. doi: 10.1007/s00438-019-01631-2. 31807910

[9] Liu J, Huang J, Guo H, Lan L, Wang H, Xu Y, Yang X, Li W, Tong H, Xiao Y, et al. The conserved and unique genetic architecture of kernel size and weight in maize and rice. Plant Physiol. 2017;175(2):774–785. doi: 10.1104/pp.17.00708. 28811335 PMC5619898

[10] Huang J, Lu G, Liu L, Raihan MS, Xu J, Jian L, Zhao L, Tran TM, Zhang Q, Liu J, et al. The kernel size-related quantitative trait locus qKW9 encodes a pentatricopeptide repeat protein that affects photosynthesis and Grain filling. Plant Physiol. 2020;183(4):1696–1709. doi: 10.1104/pp.20.00374. 32482908 PMC7401109

[11] Zuo Y, Feng F, Qi W, Song R. Dek42 encodes an RNA‐binding protein that affects alternative pre‐mRNA splicing and maize kernel development. J Integr Plant Biol. 2019;61(6):728–748. doi: 10.1111/jipb.12798. 30839161

[12] Chen Q, Zhang J, Wang J, Xie Y, Cui Y, Du X, Li L, Fu J, Liu Y, Wang J, et al. Small kernel 501 (smk501) encodes the RUBylation activating enzyme E1 subunit ECR1 (E1 C‐TERMINAL RELATED 1) and is essential for multiple aspects of cellular events during kernel development in maize. The New Phytol. 2021;230(6):2337–2354. doi: 10.1111/nph.17354. 33749863

[13] Feng J, Liu PF, Tu L, Gao Y, Guo XY, Wang AG, Zhu YF, Wu X, Chen ZH. QTL mapping and candidate gene analysis of maize kernel related traits. Seed. 2023;42(1):18–24. doi: 10.16590/j.cnki.1001-4705.

[14] Wang Q, Feng F, Zhang KH, He YH, Qi W, Ma ZY, Song RT. ZmICE1a regulates the defence–storage trade-off in maize endosperm nature plants. Nat Plants. 2024;10(12):1999–2013. doi: 10.1038/s41477-024-01845-2. 39604637

[15] Li H, Ribaut J-M, Li Z, Wang J. Inclusive composite interval mapping (ICIM) for digenic epistasis of quantitative traits in biparental populations. TAG Theor Appl Genet Theoretische Angew Genetik. 2008;116(2):243–260. doi: 10.1007/s00122-007-0663-5. 17985112

[16] Nleya T, Chungu C, Kleinjan J. Chapter 5: Corn growth and development. In: iGrow Corn: Best management practices. (CA): SDSU; 2016;23–38.

[17] Clegg KM. The application of the anthrone reagent to the estimation of starch in cereals. J Sci Food Agric. 1956;7(1):40–44. doi: 10.1002/jsfa.2740070108.

[18] Mu H, Chen J, Huang W, Huang G, Deng M, Hong S, Ai P, Gao C, Zhou H. OmicShare tools: a zero‐code interactive online platform for biological data analysis and visualization. iMeta. 2024;3(5). doi: 10.1002/imt2.228. PMC11488081 39429881

[19] Armstrong RA, Eperjesi F, Gilmartin B. The application of analysis of variance (Anova)to different experimental designs in optometry. Ophthalmic Physiol. 2002;22(3):248–256. doi: 10.1046/j.1475-1313.2002.00020.x. 12090640

[20] Pocock SJ, Stone GW. The nature of the p value. N Engl J Med. 2016;375:2205. doi: 10.1056/NEJMc1612970. 27959757

[21] Ali MM, Alam SM, Anwar R, Ali S, Shi M, Liang D, Lin Z, Chen F. Genome-wide identification, characterization and expression profiling of aluminum-activated malate transporters in Eriobotrya japonica Lindl. Horticulturae. 2021;7(11):441. doi: 10.3390/horticulturae7110441.

[22] Baum K, Gröning B, Meier I. Improved ballistic transient transformation conditions for tomato fruit allow identification of organ‐specific contributions of I‐box and G‐box to the RBCS2 promoter activity. Plant J. 1997;12(2):463–469. doi: 10.1046/j.1365-313X.1997.12020463.x. 9301095

[23] Sadeghnezhad E, Askari H, Soltani S, Honarvar F. Identification and distribution of anaerobic responsive elements (AREs) in genes functional categorization of Arabidopsis thaliana. J Appl Psychol Appl Biotechnol Reports. 2014;4:135–1411.

[24] Castelli S, Mascheretti I, Cosentino C, Lazzari B, Pirona R, Ceriotti A, Viotti A, Lauria M, Coleman CE. Uniparental and transgressive expression of α-zeins in maize endosperm of o2 hybrid lines. PLOS ONE. 2018;13(11):e0206993. doi: 10.1371/journal.pone.0206993. 30439980 PMC6237297

[25] Liu Y, Zhou W, He M, Sui J, Tian X, Guan Q, Yu X, Li K, Bu Q, Li X, et al. Comprehensive analysis of stress-activated protein kinase genes (OsSapks) in rice flowering time. Planta. 2024;259(6):149. doi: 10.1007/s00425-024-04431-0. 38724681

[26] Lou D, Lu S, Chen Z, Lin Y, Yu D, Yang X. Molecular characterization reveals that OsSAPK3 improves drought tolerance and grain yield in rice. BMC Plant Biol. 2023;23(1):53. doi: 10.1186/s12870-023-04071-8. 36694135 PMC9872327

[27] Feng F, Qi W, Lv Y, Yan S, Xu L, Yang W, Yuan Y, Chen Y, Zhao H, Song R. OPAQUE11 is a central hub of the regulatory network for maize endosperm development and nutrient metabolism. Plant Cell. 2018;30(2):375–396. doi: 10.1105/tpc.17.00616. 29436476 PMC5868688

[28] Grimald F, Rogniaux H, James MG, Myers AM, Planchot V. Proteome and phosphoproteome analysis of starch granule-associated proteins from normal maize and mutants affected in starch biosynthesis. J Exp Botany. 2008;59(12):3395–3406. doi: 10.1093/jxb/ern198. 18653693 PMC2529236

[29] Liu F, Makhmoudova A, Lee EA, Wait R, Emes MJ, Tetlow IJ. The amylose extender mutant of maize conditions novel protein-protein interactions between starch biosynthetic enzymes in amyloplasts. J Exp Botany. 2009;60(15):4423–4440. doi: 10.1093/jxb/erp297. 19805395

[30] Shoaib N, Mughal N, Liu L, Raza A, Shen L, Yu G. Site-directed mutations at phosphorylation sites in Zea mays PHO1 reveal modulation of enzymatic activity by phosphorylation at S566 in the L80 region. Plants. 2023;12(18):3205. doi: 10.3390/plants12183205. 37765369 PMC10536461

[31] Lou D, Chen Z, Yu D, Yang X. SAPK2 contributes to rice yield by modulating nitrogen metabolic processes under reproductive stage drought stress. Rice. 2020;13(1):35. doi: 10.1186/s12284-020-00395-3. 32514747 PMC7280414

[32] Luo L, Zhang Y, Xu G, Takahashi H. How does nitrogen shape plant architecture? J Exp Botany. 2020;71(15):4415–4427. doi: 10.1093/jxb/eraa187. 32279073 PMC7475096

[33] Jiang H, Shi Y, Liu J, Li Z, Fu D, Wu S, Li M, Yang Z, Shi Y, Lai J, et al. Natural polymorphism of ZmICE1a contributes to amino acid metabolism that impacts cold tolerance in maize. Nat Plants. 2022;8(10):1176–1190. doi: 10.1038/s41477-022-01254-3. 36241735

[34] Yang L. Bridging the perception: ICE1 links cold sensing and salicylic acid signaling. Plant Cell. 2024;36(7):2457–2458. doi: 10.1093/plcell/koae115. 38598662 PMC11218770

