# Supplementary figures and images for: Cloning and function analysis of ZmICE1a, a contributor to the melioration of maize kernel traits
Source: Plant Signal Behav. 2025 Jul 2;20(1):2521320. doi: 10.1080/15592324.2025.2521320 (PMC12233868; doi:10.1080/15592324.2025.2521320)

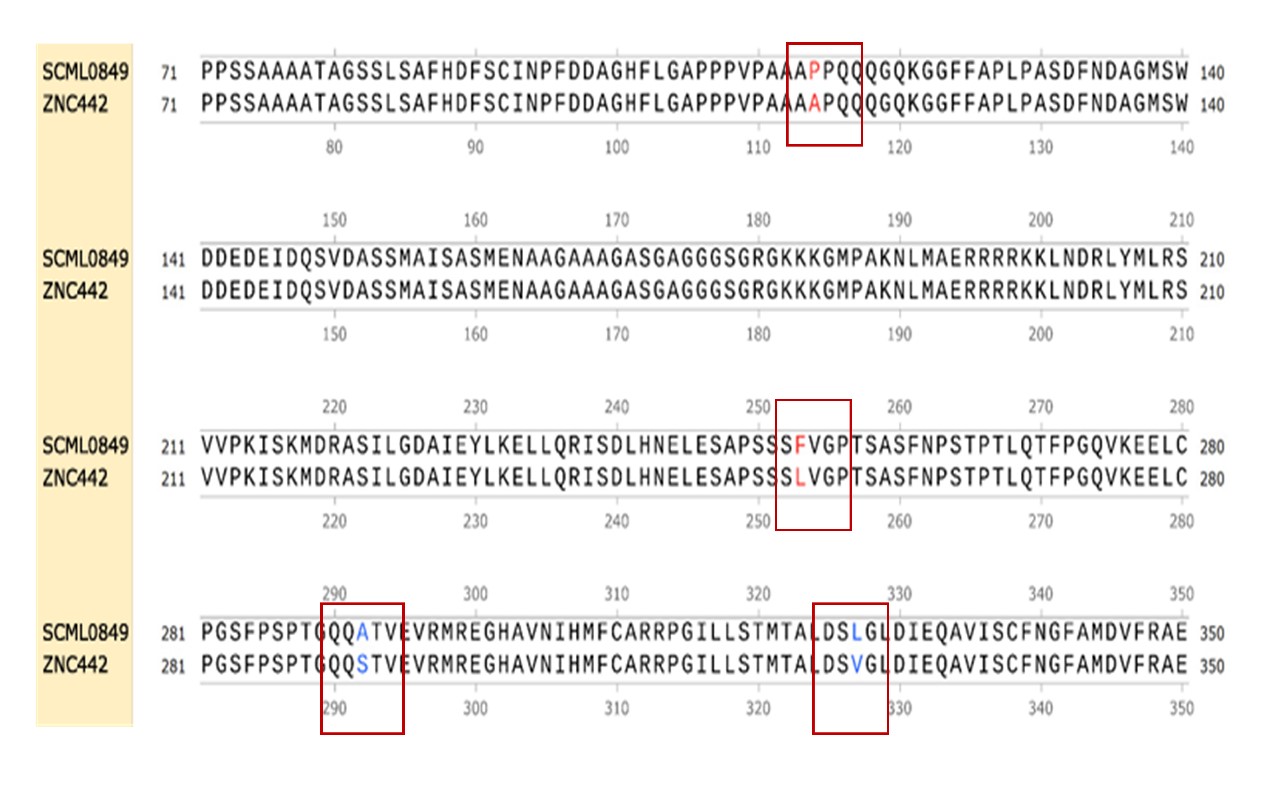

Supplement: Supplemental Material [file KPSB_A_2521320_SM0144.zip › Supplementary Figure 3.jpg]

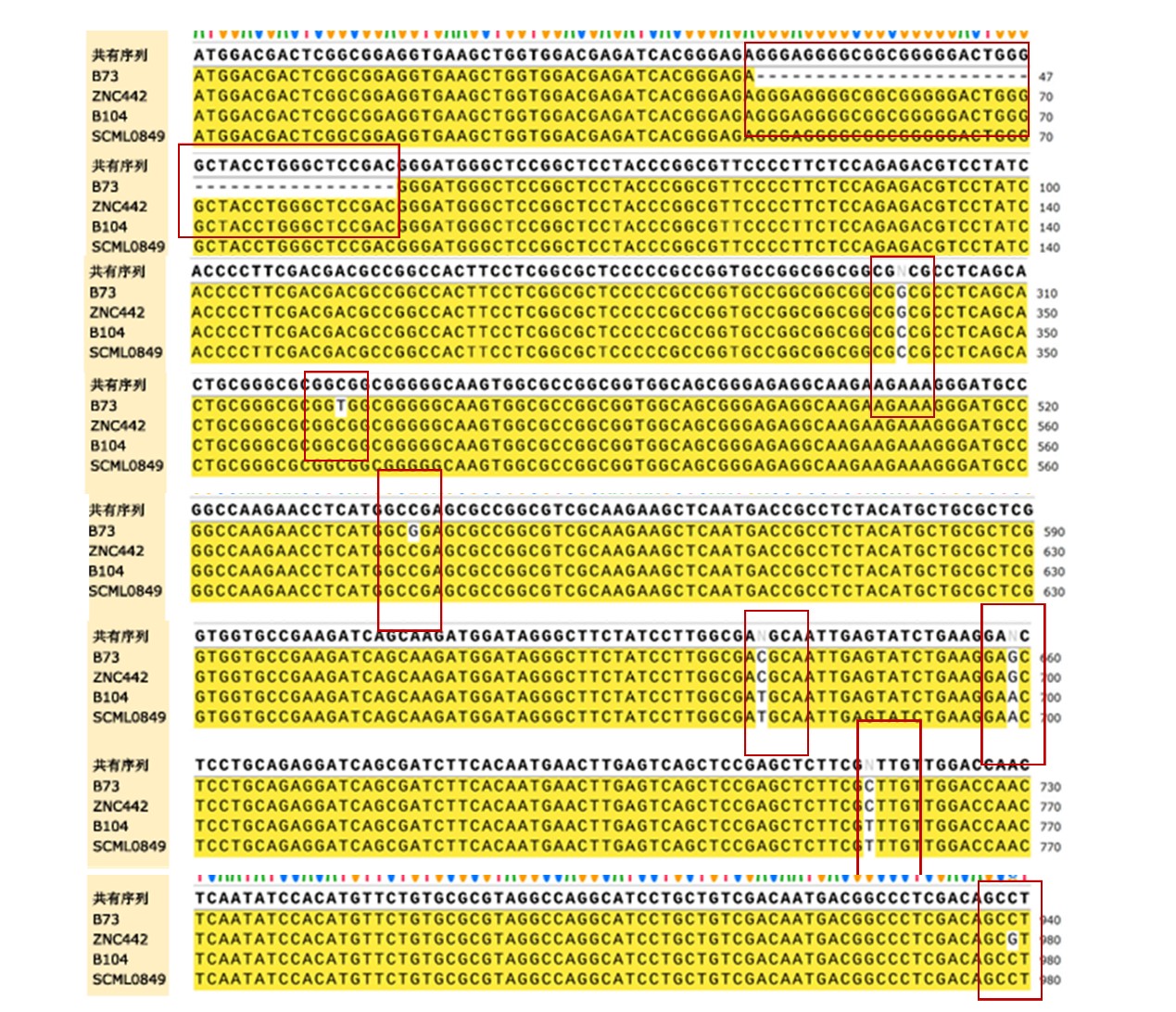

Supplement: Supplemental Material [file KPSB_A_2521320_SM0144.zip › Supplementary Figure 2.jpg]

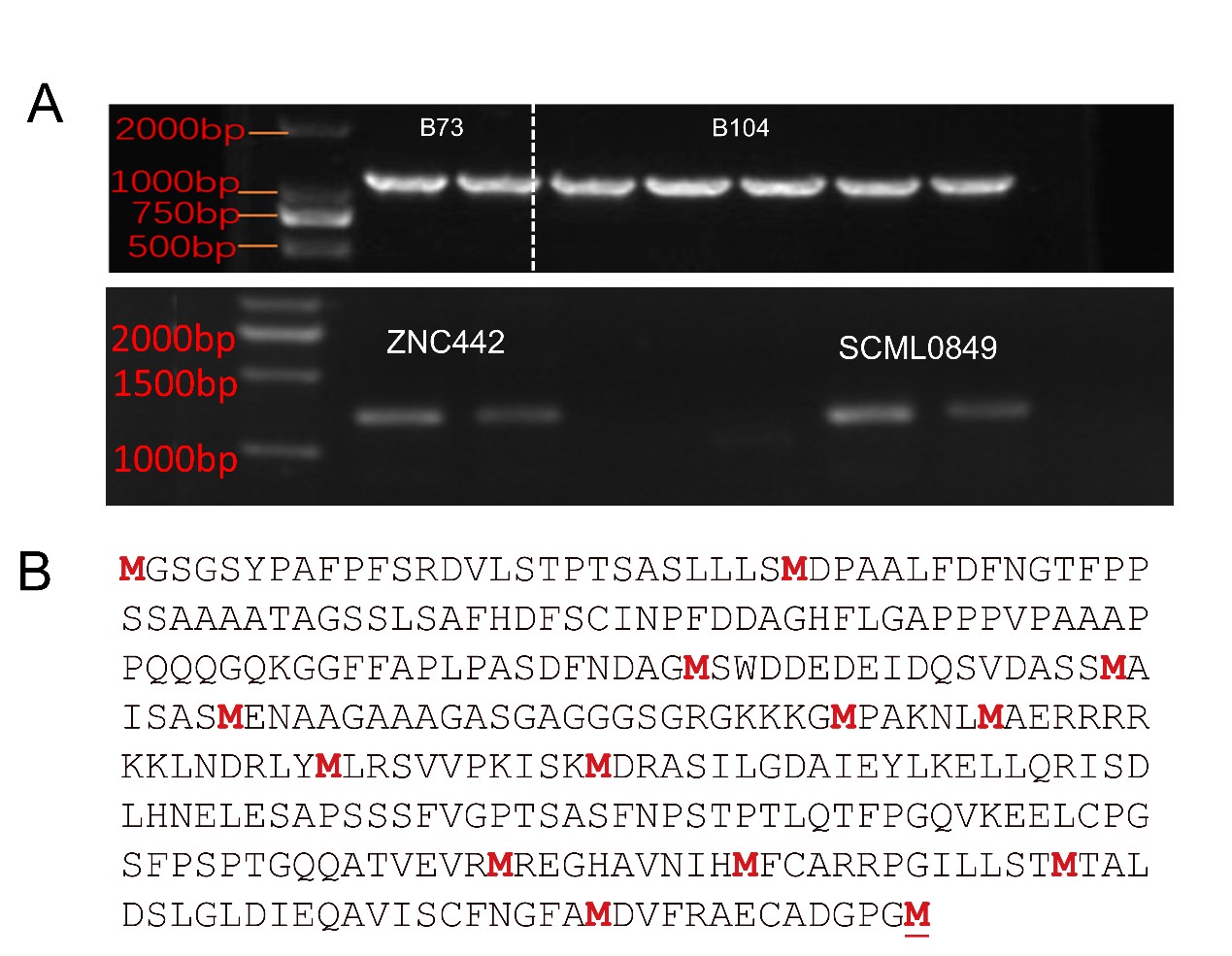

Supplement: Supplemental Material [file KPSB_A_2521320_SM0144.zip › Supplementary Figure 1.jpg]

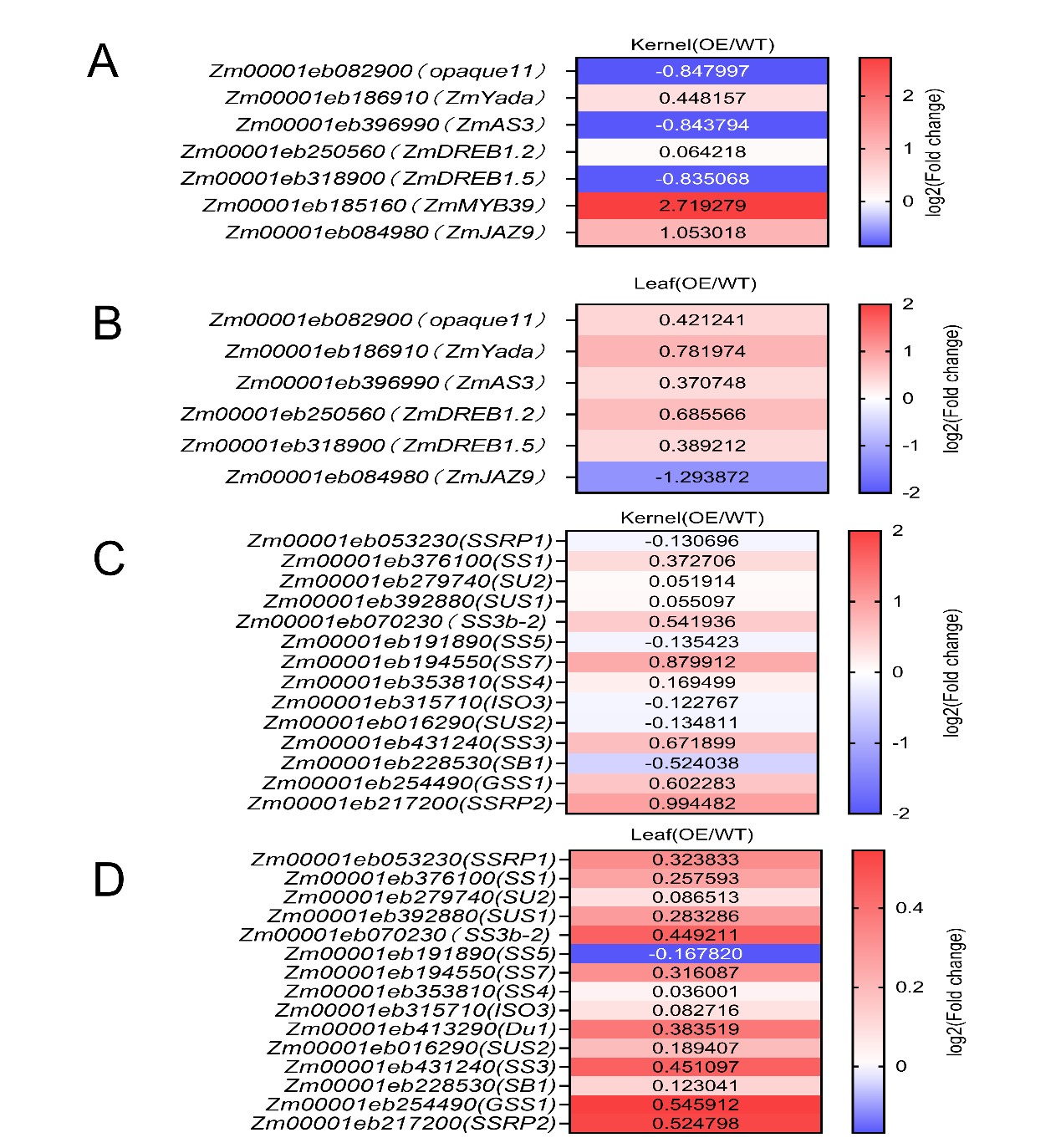

Supplement: Supplemental Material [file KPSB_A_2521320_SM0144.zip › Supplementary Figure 5.jpg]

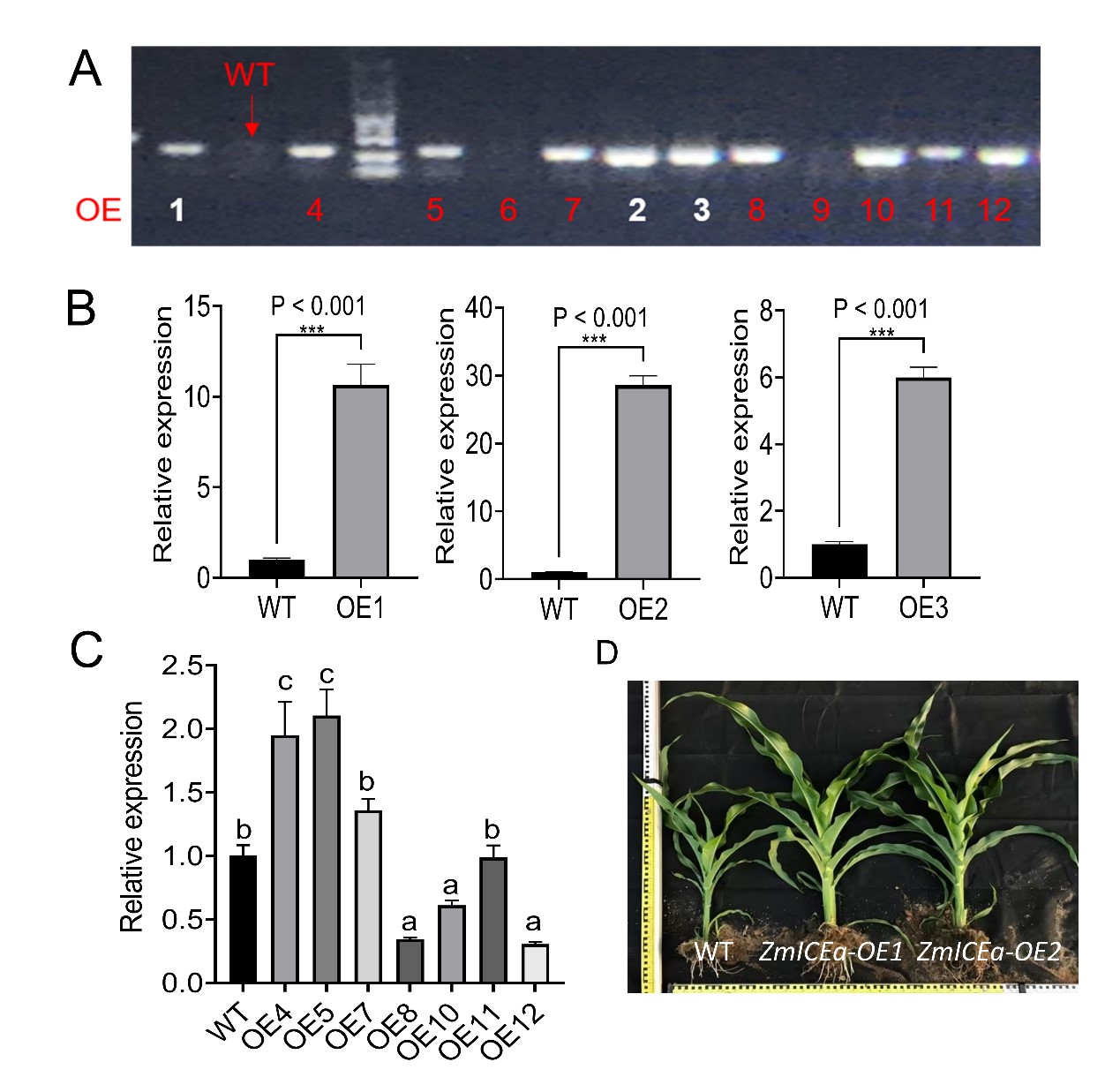

Supplement: Supplemental Material [file KPSB_A_2521320_SM0144.zip › Supplementary Figure 4.jpg]
